# SENP1 desensitizes hypoxic ovarian cancer cells to cisplatin by up-regulating HIF-1α

**DOI:** 10.1038/srep16396

**Published:** 2015-11-09

**Authors:** Qilin Ao, Wenjing Su, Shuang Guo, Lei Cai, Lei Huang

**Affiliations:** 1 Institute of Pathology of Tongji Hospital and Department of Pathology, Tongji Medical College, Huazhong University of Science and Technology, Wuhan 430030, China; 2 Department of Pathology, Shandong Provincial Hospital, Shandong University, Jinan 250021, China; 3 Department of Pathology, Union Hospital, Tongji Medical College, Huazhong University of Science and Technology, Wuhan 430030, China; 4 Department of Obstetrics & Gynecology, Central Hospital of Wuhan, Wuhan 430014, China

## Abstract

Hypoxia-inducible factor 1 alpha (HIF-1α) is closely related to chemoresistance of ovarian cancers. Although it is reported that HIF-1α can be regulated by Sentrin/SUMO-specific protease 1 (SENP1), the effects of SENP1 on HIF-1α is still controversial. In this study, we identified that SENP1 positively regulated the expression of HIF-1α by deSUMOylation and weakened the sensitivity of hypoxic ovarian cancer cells to cisplatin. These results indicate that SENP1 is a positive regulator of HIF-1α and plays a negative role in ovarian cancer chemotherapy.

Ovarian cancer is the most fatal malignancy in the female reproductive system and the fifth leading cause of death from cancer in women^1^. In the past few years, the combination of cytoreductive surgery and chemotherapy has increased the survival time of ovarian cancer patients. However, many cases relapsed within 3 years and became resistant to the first-line cisplatin-centered chemotherapy^2^. The unsatisfactory results encourage further investigation of the underlying mechanism.

The transcription factor hypoxia-inducible factor-1 (HIF-1) activates the transcription of more than 100 genes involved in cancer biology such as proliferation, apoptosis and angiogenesis^3^, and maintains major roles in cellular responses to hypoxia which cause tumor chemoresistance. HIF-1 exists as a αβ heterodimer, the activation of which is dependent on stabilization of the O_2_-dependent α subunit. HIF-1α was overexpressed in more than 70% solid tumors and closely correlated with the poor prognosis of tumors^4^. HIF-1α also plays important roles in ovarian cancer chemoresistance^5^,^6^, which confers extra significance to correlated studies.

The research about HIF-1α degradation has mainly concentrated on the pVHL-mediated ubiquitin–proteasome pathway. These years, modifications other than hydroxylation such as SUMOylation and deSUMOylation have been discovered to participate in HIF-1α regulation. SENP1, a nuclear SUMO protease, may play an important role in HIF-1α deSUMOylation^7^.

It is recognized that Cobalt chloride (CoCl_2_) promotes a response similar to hypoxia because cobalt iron can replace iron from the iron-binding center of specific prolyl hydroxylases and inactivate hydroxylation activity. CoCl_2_ has been widely used to simulate hypoxia condition in numerous systems including ovarian cancer.

In this study, we mimicked a hypoxic model of ovarian cancer with CoCl_2_, investigated the effects of SENP1 on HIF-1α and the internal mechanisms, and detected the effects of SENP1 on ovarian cancer chemosensitivity. The results may have important implications for the potential use of SENP1 regulation in HIF-1α inhibition and for improving chemosensitivity of ovarian cancer cells to cisplatin.

## Results

### The establishment of a hypoxia model in ovarian cancer SKOV3 cells

**Figure 1:**
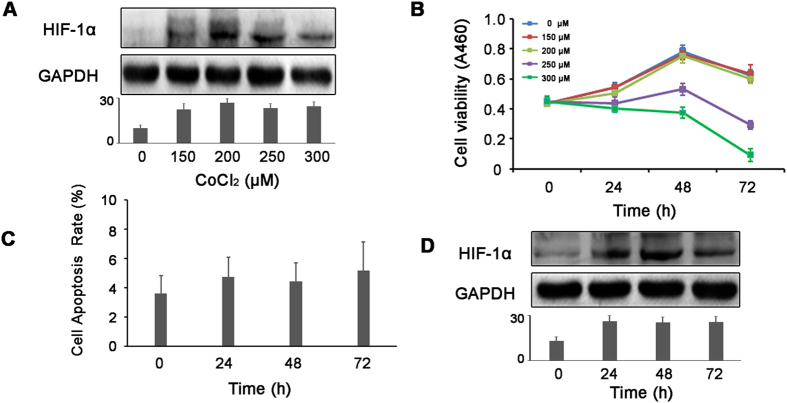
200 μM CoCl_2_ effectively induces hypoxia in SKOV3 cells. (**A**) SKOV3 cells were treated with gradient concentrations of CoCl_2_ for 48 h, and the expression of HIF-1α was examined by western blot. (**B**) CCK8 assay showed that CoCl_2_ with the concentration of 150 uM and 200 uM had no significant impact on the proliferation of SKOV3 cells. (**C**) SKOV3 cells were treated with 200 uM CoCl_2_ for different durations. Flow cytometry showed no significant difference in cell apoptosis rate between the groups. (**D**) SKOV3 cells were treated with 200 μM CoCl_2_ for 24 h, 48 h and 72 h. The expression of HIF1α was examined by western blot.

First, we tried to find out an appropriate dose of CoCl_2_ which induced hypoxia but did not impact cell viability. The ovarian cancer SKOV3 cells were treated with gradient concentrations (from 150 μM to 300 μM) of CoCl_2_. As shown in Fig. 1A, HIF-1α protein levels increased upon CoCl_2_ treatment, while the mRNA levels not (data not shown). CCK8 and FCM showed that 150 μM or 200 μM CoCl_2_ had no significant impact on the basic proliferation and cell apoptosis rate of SKOV3 (*P* < 0.05) (Fig. 1B,C). Also, 200 μM CoCl_2_ maintained high HIF-1α protein levels in SKOV3 cells in 72 hours (Fig. 1D). Based on the above data, 200 μM CoCl_2_ was chosen to mimic ovarian cancer hypoxia in the following studies.

### Hypoxia decreases sensitivity of SKOV3 cells to cisplatin

**Figure 2:**
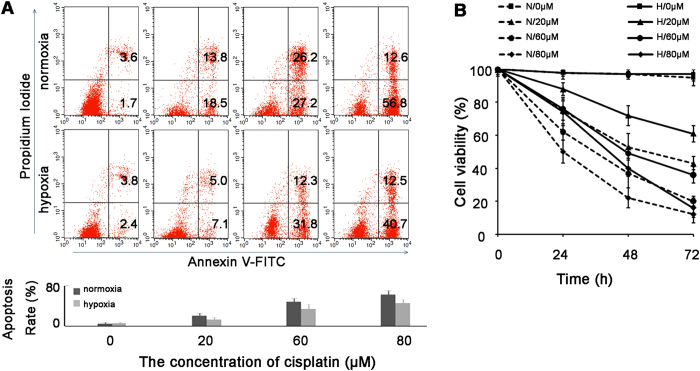
Hypoxia decreases sensitivity of SKOV3 cells to cisplatin. (**A**) SKOV3 cells were treated with the indicated concentrations of cisplatin for 48 h in both normoxia and hypoxia. Flow cytometry showed that the cell apoptosis rates in hypoxia were significantly lower than those in normoxia with the same cisplatin concentration (*P* < 0.01). (**B**) SKOV3 cells were treated with the indicated concentrations of cisplatin in both normoxia and hypoxia (N = normoxia, H = hypoxia). Cell viability was analyzed by CCK8 assay and the points represented averages of three independent experiments.

FCM and CCK8 assay were used to measure the sensitivity of SKOV3 to cisplatin. As shown in Fig. 2A, cisplatin induced SKOV3 apoptosis in a dose-dependent manner in both normoxia and hypoxia. However, the cell apoptosis rates in normoxia (32.3%, 53.4% and 69.4%) were significantly higher than those in hypoxia (12.1%, 44.1% and 53.2%) with 20 μM, 60 μM and 80 μM cisplatin treatments in turn (*P* < 0.05), which indicated a poorer reactivity of hypoxic cells to cisplatin. CCK8 assay also showed that hypoxic cells (solid lines) had poorer response to cisplatin than those in normoxia (dotted lines) (*P* < 0.05) (Fig. 2B).

### SENP1 positively regulated HIF-1α in SKOV3 cells

**Figure 3:**
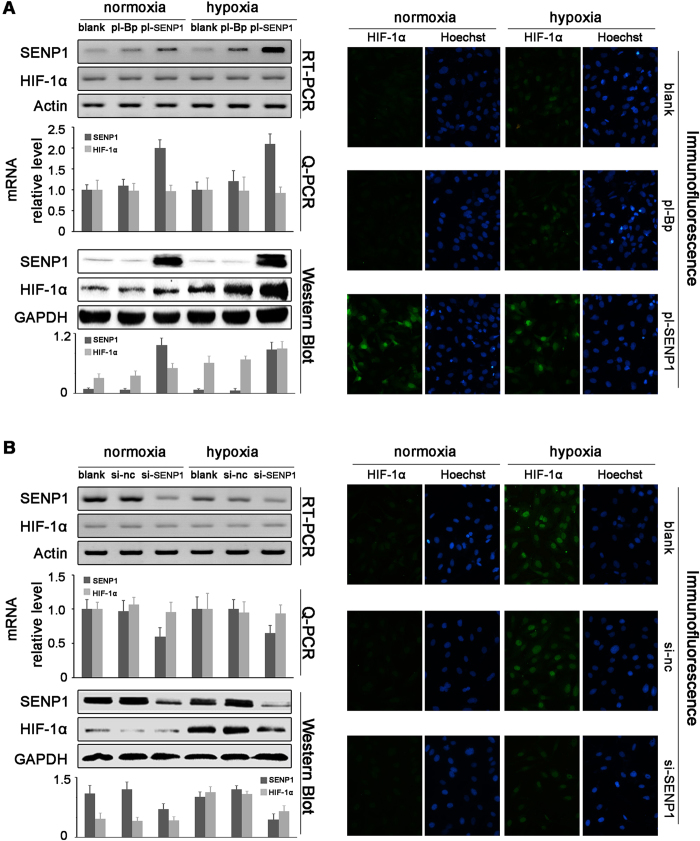
SENP1 positively regulates the expression of HIF-1α. (**A**) SKOV3 cells were transfected with SENP1 expression plasmid (pl-SENP1; pl-Bp as negative control). RT-PCR and Q-PCR showed that pl-SENP1 had no impact on HIF-1α mRNA level. Western blot and immunofluorescence (200×) showed that pl-SENP1 upregulated the protein levels of HIF-1α in both normoxia and hypoxia. (**B**) SKOV3 cells were transfected with siRNA aiming at SENP1 (si-SENP1; si-nc as negative control). RT-PCR and Q-PCR showed that si-SENP1 had no significant impact on HIF-1α mRNA level. Western blot and immunofluorescence (200×) showed that si-SENP1 downregulated the protein levels of HIF-1α in hypoxia but normoxia.

To detect the effect of SENP1 on HIF-1α, SKOV3 cells were transfected with SENP1 plasmids (pl-SENP1) in the presence (hypoxia) or absence (normoxia) of 200 μl CoCl_2_ (Fig. 3A). RT-PCR and Q-PCR showed that SENP1 overexpression did not impact the mRNA levels of HIF-1α in either normoxia or hypoxia. However, western blot showed that pl-SENP1 significantly increased HIF-1α protein levels in both normoxia and hypoxia. Immunofluorescence showed that CoCl_2_ treatment could induce HIF-1α expression in SKOV3 cells. Also, pl-SENP1 enhanced HIF-1α accumulation in nuclei in both normoxia and hypoxia, indicating that SENP1 enhanced the expression and activity of HIF-1α.

Furthermore, we used siRNA aiming at SENP1 (si-SENP1) to inhibit SENP1 and to see its effects on HIF-1α. As shown in Fig. 3B, si-SENP1 decreased the protein but mRNA level of HIF-1α especially in hypoxia. Immunofluorescence showed that si-SENP1 weakened HIF-1α accumulation in nuclei in hypoxia, indicating that SENP1 positively regulated the expression of HIF-1α. However, this effect was not obvious in normoxia, which might be explained by the basal low HIF-1α level in normoxia.

### SENP1 was essential for the deSUMOylation of HIF-1α

**Figure 4:**
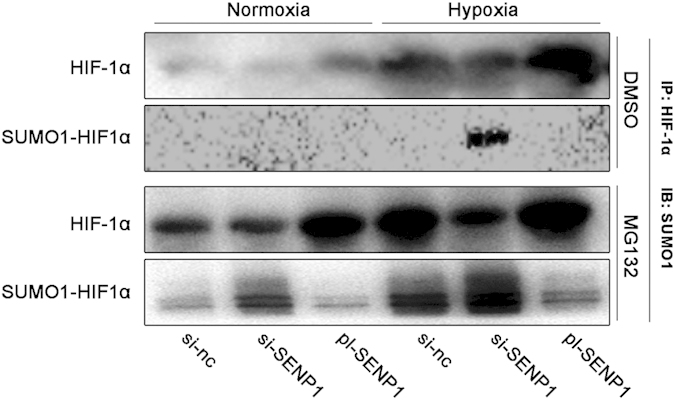
SENP1 maintains HIF-1α stability through desumoylation. HIF-1α was first immunoprecipitated (IP) with HIF-1α antibody from cell nuclei lysates and the precipitates were then immunoblotted (IB) with SUMO-1 antibody. The transfection of si-SENP1 induced increased SUMO1-HIF1α combination and pl-SENP1 caused the opposite effect especially in hypoxia. Treatment with MG132 (50 μM, 3 hours) enhanced those effects.

The previous results showed that SENP1 positively regulated HIF-1α protein level in hypoxic SKOV3 cells. Subsequently, co-immunofluorenscence was used to explore the mechanisms how SENP1 regulated HIF-1α. As shown in Fig. 4, SUMOylated HIF-1α can only be detected in hypoxic SKOV3 cells transfected with si-SENP1, which confirmed that SENP1 deSUMOylated HIF-1α. However, MG132 evidently increased HIF-1α SUMOylation, suggesting that the level of SUMOylated HIF-1α was controlled by a proteasome-dependent mechanism. Taken together, these results suggested that SENP1 maintained HIF-1α stability through desumoylation, which was followed by the interdiction of a proteasome-mediated HIF-1α degradation.

### SENP1 desensitized SKOV3 cells to cisplatin in hypoxia

**Figure 5:**
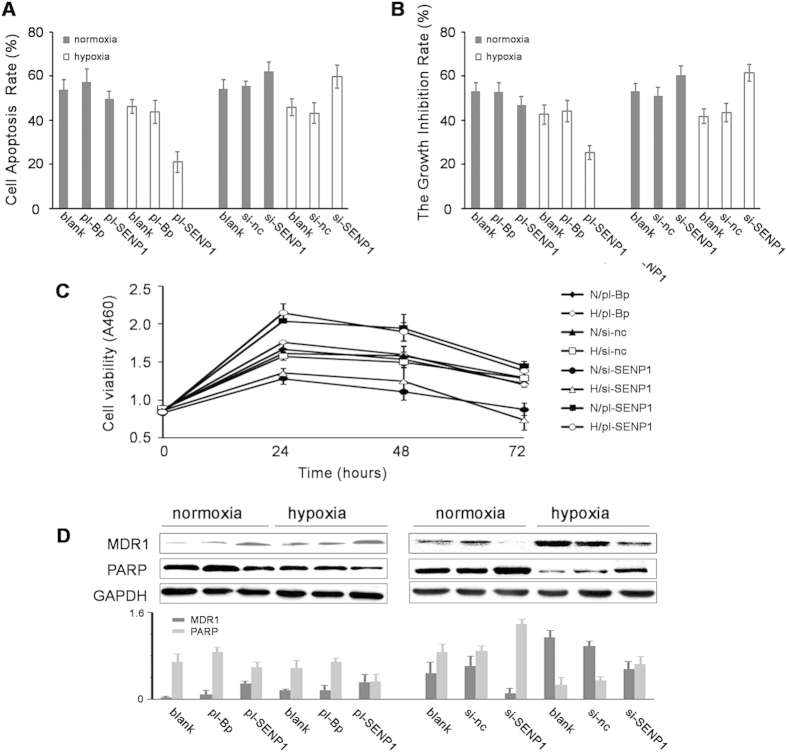
SENP1 decreases the sensitivity of SKOV3 cells to cisplatin in hypoxia. SKOV3 cells transfected with pl-SENP1 or si-SENP1 were treated with 60 μM cisplatin for 48 hours. (**A**) Flow cytometry showed that pl-SENP1 significantly decreased the cell apoptosis rate while si-SENP1 significantly upregulated the cell apoptosis rate in hypoxia (*P* < 0.05). However, this phenomenon was not obvious in normoxia. Trypan blue exclusion assay (**B**) and CCK8 assay (**C**), N = normoxia H = hypoxia) showed the similar results as in (**A,D**) Western blot showed that SENP1 upregulated the protein level of MDR1 and downregulated the protein level of PARP especially in hypoxia.

Considering the close relationship between HIF-1α and chemosensitivity, we subsequently examined the effect of SENP1 on the sensitivity of SKOV3 cells to cisplatin. FCM showed that in hypoxia, pl-SENP1 decreased cisplatin-induced apoptosis from 46.3% to 21.0% and si-SENP1 enhanced cisplatin-induced apoptosis from 45.8% to 59.6% (Fig. 5A). Trypan blue exclusion assay showed that in hypoxia, pl-SENP1 decreased cisplatin-induced cell death from 42.6% to 25.4% and si-SENP1 enhanced cisplatin-induced cell death from 41.8% to 61.4% (Fig. 5B). However, it seems that pl-SENP1 had no significant impact on cisplatin-induced cell apoptosis (or cell death) in normoxia. In Fig. 5C, CCK8 showed similar results with trypan blue exclusion assay. We also detected the impacts of SENP1 on MDR1 (a well-known HIF-1 target) and PARP (an immediate indicator to DNA damage). As shown in Fig. 5D, SENP1 positively regulated the expression of MDR1 and abolished the level of the apoptosis related gene PARP especially in hypoxia. These effects may provide the mechanistic basis for chemosensitization by inhibiting SENP1.

## Discussion

Among the numerous factors contributing to tumor malignant behaviors, hypoxia and HIF-1α overexpression have attracted much attention and contribute a lot to solid tumor chemoresistance^8^,^9^. In the present study, we for the first time confirmed that SENP1 inhibition can sensitize ovarian cancer cells to cisplatin in hypoxia by inhibiting HIF-1α.

The microenvironment of rapidly growing tumors is associated with increased energy demand and diminished vascular supply, resulting in focal areas of chronic hypoxia. Hypoxia inhibits proteasome-dependent degradation of HIF-1α and results in HIF-1αstabilization. HIF-1α overexpression has been observed in a series of solid tumors such as glioma^10^, renal cancer^11^, prostate cancer^12^ and lung cancer^13^. In ovarian cancer, HIF-1α plays pivotal roles in chemoresistance by arresting cell cycle at G0/G1 phase^5^. Also, HIF-1α hyperfunction is closely correlated with treatment failure and patient mortality in early-stage cervical cancer^14^.

HIF-1α dimerizes with HIF-1β and activates target genes by binding to hypoxia responsive element (HRE) within their promoters. HIF-1 induces the expression of a number of hypoxia-responsive genes such as the well-known multi-drug resistance 1 (MDR1), EPO and VEGF^15^,^16^,^17^. HIF-1 also inhibits cell death by mediating expression of the anti-apoptotic BIR5/survivin and Mcl-1 genes, although the pro-apoptotic genes BNIP3 and NIX are also HIF-1 targets^18^,^19^,^20^. The broad impact of the HIF-1 transcription factor on gene expression makes it a key element in regulation of cell survival, apoptosis, cell motility, cytoskeletal structure, cell adhesion, erythropoiesis, vasculature tone, epithelial homeostasis, drug resistance, energy metabolism and pH regulation. Also, the extent of hypoxia is correlated with clinical stage and treatment failure of solid tumors, although the precise role remains highly controversial.

The most classic studies about HIF-1α stability focus on the pVHL-mediated ubiquitin–proteasome pathway^21^,^22^. During normoxia, HIF-1α locates in the cytoplasm and is hydroxylated by the prolyl 4-hydroxylases (PHD) enzyme, then binds to VHL, and becomes ubiquitinated and degraded by the proteasome. However, proline hydroxylation occurs inefficiently in hypoxia, thus allowing HIF-1α to escape proteasomal degradation. Besides, there are also modifications other than hydroxylation, such as acetylation, phosphorylation and S-nitrosation, which all regulate HIF-1α stability^23^.

Recently, SUMOylation and deSUMOylation cycle was confirmed to involve in the regulation of HIF-1α in hypoxia^24^. Hypoxia induces nuclear translocation and subsequent SUMOylation of HIF-1α, with the ligating enzyme E3 playing a major role in that process^25^,^26^. SUMOylation of HIF-1α, which binds to VHL in a hydroxyl proline-independent manner, leading to ubiquitination and proteasomal degradation in the nucleus. However, SUMO conjugation is a dynamic process, in that it can be readily reversed by a family of Sentrin/SUMO-specific proteases (SENPs). Among them, SENP1 is reported to be induced by hypoxia, and hypoxia-induced stabilization of HIF-1α is dependent on SENP1, which deconjugates hypoxia-induced SUMOylated HIF-1α and prevents the degradation mediated by the ubiquitination of SUMOylated HIF-1^26^. However, there are also reports displaying that HIF-1α is upregulated through SUMO-1 modfication at Lys 391/Lys 477 residues, which may stabilize HIF-1α and enhance its transcriptional activity^7^. Alberto Carbia-Nagashima *et al.* reported that RSUME, a Small RWD-Containing Protein, enhanced SUMO conjugation and stabilized HIF-1α during Hypoxia^27^. These contrasting results from the previous studies may be attributed to the overexpression of SUMO-1 in the presence of SENP1.

In this study, we examined the expression of HIF-1α protein in ovarian cancer cells of SKOV3 by inhibiting or up-regulating SENP1 and found that SENP1 stabilized HIF-1α protein in hypoxia through desumoylation. What is more, sumoylated HIF-1αdegraded via proteasome pathway. These results coincided with the conclusion of Cheng *et al.*^26^. In addition, we found that SENP1 desensitized SKOV3 cells to cisplatin, positively regulated the expression of HIF-1 target MDR1, and decreased the level of the apoptosis associated gene PARP in hypoxia.

In summary, the results of this study indicated the positive regulation of SENP1 on HIF-1α. Also, our data provide the possibility that by combing SENP1 inhibition with standard chemotherapy in ovarian cancer tumor resistance may be reduced.

## Methods

### Cell line and general reagents

The human ovarian cancer cell line SKOV3 was from the American Type Culture Collection and maintained in RPMI1640 with 10% fetal calf serum (Life Technologies, Carlsbad, CA). Cisplatin and CoCl_2_ were obtained from Sigma Chemical Inc. (St. Louis, Missouri, USA).

### Reverse transcription-PCR and real-time quantitative PCR

RT-PCR and real-time quantitative PCR were performed as previously described^28^. The PCR primers used are as follows: HIF-1α (5′-CGT TCC TTC GAT CAG TTG TC-3′, 5′-TCA GTG GTG GCA GTG GTA GT-3′), SENP1 (5′-CAT CTG TCT TAC CTT CCT-3′, 5′-TCA CTA ACT TCT ACC TTG T-3′), β-actin (5′-CTG GCA CCA CAC CTT CTA CAA TG-3′, 5′-CCT CGT AGA TGG GCA CAG TGT G-3′).

### Western blot

After appropriate treatment, protein extracts were loaded onto a 10% SDS-polyacrylamide gel, electrophoresed, transferred to a PVDF membrane and incubated with antibodies. The primary antibodies used were as follows: HIF-1α (mouse monoclonal, 1:500, NOVUS, Colorado, USA), SENP1 (rabbit monoclonal, 1:500, Epitomics, Burlingame, CA), SUMO-1 (rabbit monoclonal, 1:1,000, Epitomics, Burlingame, CA), MDR1 (rabbit polyclonal, 1:5,00, NOVUS, Colorado, USA), PARP (rabbit polyclonal, 1:5,00, Cell Signaling Technology, Danvers, MA), GAPDH (mouse monoclonal, 1:10,000, Kangcheng, Shanghai, China).

### Cell Counting Kit-8 (CCK8) assay

100 μl cell suspensions (3000 cells/well) were dispensed in 96-well plates, pre-incubated for 24 hours in an incubator (humidified atmosphere, 37 °C, 5% CO_2_) and treated as needed. Subsequently, 20 μl CCK8 (Roche Diagnosis, Mannheim, Germany) was added to each well, incubated for 2 h. The absorbance at 450 nm (A450) was examined using Epoch Multi-Volume Spectrophotometer System (Biotek, Vermont, USA).

### Flow cytometric analysis

Cell apoptosis was detected by Annexin V-FITC Apoptosis Detection Kit (KeyGen Biotech, Nanjing, China) as previously described^6^. In brief, 2 × 10^5^ cells were collected, washed twice with PBS, resuspended in 500 μl binding buffer and incubated with 5 μl Annexin V-FITC and 5 μl propidium iodide (PI) for 10 min before analysis using a FACS Calibur flow cytometer (BD Biosciences, USA).

### Small interfering RNA transfection

Lipofectamine TM 2000 (Invitrogen, Carlsbad, CA, USA) were used to transfect siRNAs into SKOV3 cells. The siRNA sequence used for SENP1 interference (si-SENP1) was as follows: 5′-CTAAACCATCTGAATTGGCTC-3′. The antisense strand of this sequence was used as nonspecific control (si-nc).

### Plasmid transfections

The X-tremeGENE HP DNA Transfection Reagent (Roche Diagnosis, Mannheim, Germany) was chosen to transfect the eukaryotic coexpression plasmid encoding SENP1 (pl-SENP1, GenScript, Nanjing, China) into SKOV3 cells, with the empty pEGFP-C1 vector as negative control (pl-Bp).

### Immunofluorescence

Cells were placed on glass coverslips in 24-well plates and were allowed to adhere overnight. Subsequently, cells were treated as needed and fixed with precooled ethanol (−20 °C) for 15 min. Then, coverslips were blocked in 5% bovine serum albumin for 1 h at 37 °C and processed for immunofluorescence with HIF-1α antibody. FITC-labeled goat anti-mouse antibody was used as secondary antibody. Hoechst 33258 was used to stain the nuclei. Coverslips were then mounted onto glass slides and examined using an inverted fluorescence microscope (Leica, Germany).

### Co-immunoprecipitation

Co-immunoprecipitation was carried out according to routine instructions. To put it briefly, cell nuclei were collected, lysed and centrifuged. Then the supernatant was transferred to new tubes and pre-incubated with protein A/G-agarose beads so as to exclude subsequent non-specific binding. Then the supernatant after centrifugation was transferred to new tubes and incubated with antibody-coupled protein A/G-agarose. Finally the protein complexes were eluted and preceded to analysis.

### Trypan blue exclusion assay

Cells were stained with 200 mg/ml trypan blue (Sigma, St. Louis, Missouri, USA). The number of viable cells was examined with microscope.

### Statistical analysis

All experiments were repeated at least thrice. PASW Statistics 18.0 software package was used for statistical analysis. All values were presented as mean ± standard deviation (SD). *P* value less than 0.05 was considered statistically significant.

## Additional Information

**How to cite this article**: Ao, Q. *et al.* SENP1 desensitizes hypoxic ovarian cancer cells to cisplatin by up-regulating HIF-1α. *Sci. Rep.* **5**, 16396; doi: 10.1038/srep16396 (2015).
